# Odontogenic carcinosarcoma of the mandible: A case report

**DOI:** 10.1002/ccr3.9554

**Published:** 2024-11-11

**Authors:** Pouyan Aminishakib, Farzaneh Mosavat, Mahsa Bayati, Ata Garajei

**Affiliations:** ^1^ Department of Oral and Maxillofacial Pathology, Vice‐Head for International Affairs, School of Dentistry Tehran University of Medical Sciences Tehran Iran; ^2^ Department of Oral and Maxillofacial Radiology, Faculty of Dentistry Tehran University of Medical Sciences Tehran Iran

**Keywords:** histopathological characteristics, malignant odontogenic tumor, mixed tumor, odontogenic carcinosarcoma, odontogenic tumor

## Abstract

**Key Clinical Message:**

Odontogenic carcinosarcoma, a rare and challenging diagnosis, was identified in a 60‐year‐old male through histopathology, revealing a biphasic neoplasm with malignant epithelial and mesenchymal components. Surgical resection is crucial for management, highlighting the importance of vigilant postoperative follow‐up to ensure early detection of any recurrence.

**Abstract:**

One rare mixed malignant odontogenic tumor is odontogenic carcinosarcoma, which comprises malignant epithelial and mesenchymal components. Diagnosing odontogenic carcinosarcoma is challenging due to its rarity and atypical clinical presentation. This study reports a 60‐year‐old male patient who presented with a painless swelling on the right side of his face and experienced facial asymmetry for 6 months, ultimately diagnosed with odontogenic carcinosarcoma. A biphasic neoplasm with malignant alterations in both epithelial and mesenchymal components was identified upon histopathological examination. MRI imaging showed an expansile multilobulated lytic lesion with cortical erosion and extraosseous extension in the posterior region of the right mandibular body and ramus. Following contrast administration, homogeneous lesion enhancement was observed, with a few small non‐enhancing necrotic areas in central parts. The patient subsequently underwent a right hemi‐mandibulectomy with resection of adjacent soft tissues and neck dissection due to lymph node involvement. The resulting defect was reconstructed using a pectoralis major flap. No recurrence or metastasis was reported during the 6‐month follow‐up, reinforcing the positive results. This case highlights the importance of recognizing odontogenic carcinosarcoma and underscores the challenges in diagnosing and managing this rare tumor. Early identification and aggressive treatment can lead to positive outcomes, as evidenced by the absence of recurrence or metastasis in this patient during the follow‐up period.

## 1 INTRODUCTION

Odontogenic tumors (OTs) encompass a spectrum of lesions with diverse clinical and histopathologic features. They are broadly categorized into malignant and benign groups.^1^, ^2^, ^3^ Odontogenic carcinosarcoma (OCS) is a rare malignancy among OTs, initially classified as a malignant OT in 1992.^4^ OCS is bi‐phasic, comprising both sarcomatous and carcinomatous components.^1^, ^2^, ^4^ Although its etiology remains relatively unclear, it has been suggested to be associated with specific benign lesions like ameloblastoma (AB), ameloblastic fibroma (AF),^5^, ^6^, ^7^ and ameloblastic fibrosarcoma (AFS).^6^, ^7^ However, due to limited reported cases, the exact mechanisms of this transformation still need to be explored.^6^, ^7^ We present a comprehensive illustration of OCS in the present case, including clinical, radiographic, immunohistochemical, and histopathologic findings.

## 2 CASE HISTORY/EXAMINATION

In July 2022, a 60‐year‐old male patient visited the Cancer Institute in Tehran, reporting a painless swelling on the right side of his face that had been present for 6 months and was accompanied by facial asymmetry. His past medical history was unremarkable, with no significant health issues before the onset of this condition. Upon physical examination, a large, purple, fluctuant mass involving the right mandibular posterior body and ramus was observed, with no other abnormalities noted, and there was no evidence of cranial nerve dysfunction.

## 3 METHODS

### 3.1 Differential diagnosis

Initial considerations included benign and malignant odontogenic tumors such as ameloblastoma (AB), Ameloblastic fibrosarcoma (AFS), and Squamous cell carcinoma, given the patient's symptoms and clinical presentation.

### 3.2 Investigations

Soft Tissue Sonography revealed a hypoechoic mass measuring 49 × 22 × 40 mm on the right side of the mandible, with cortical plate disruption.

**FIGURE 1:**
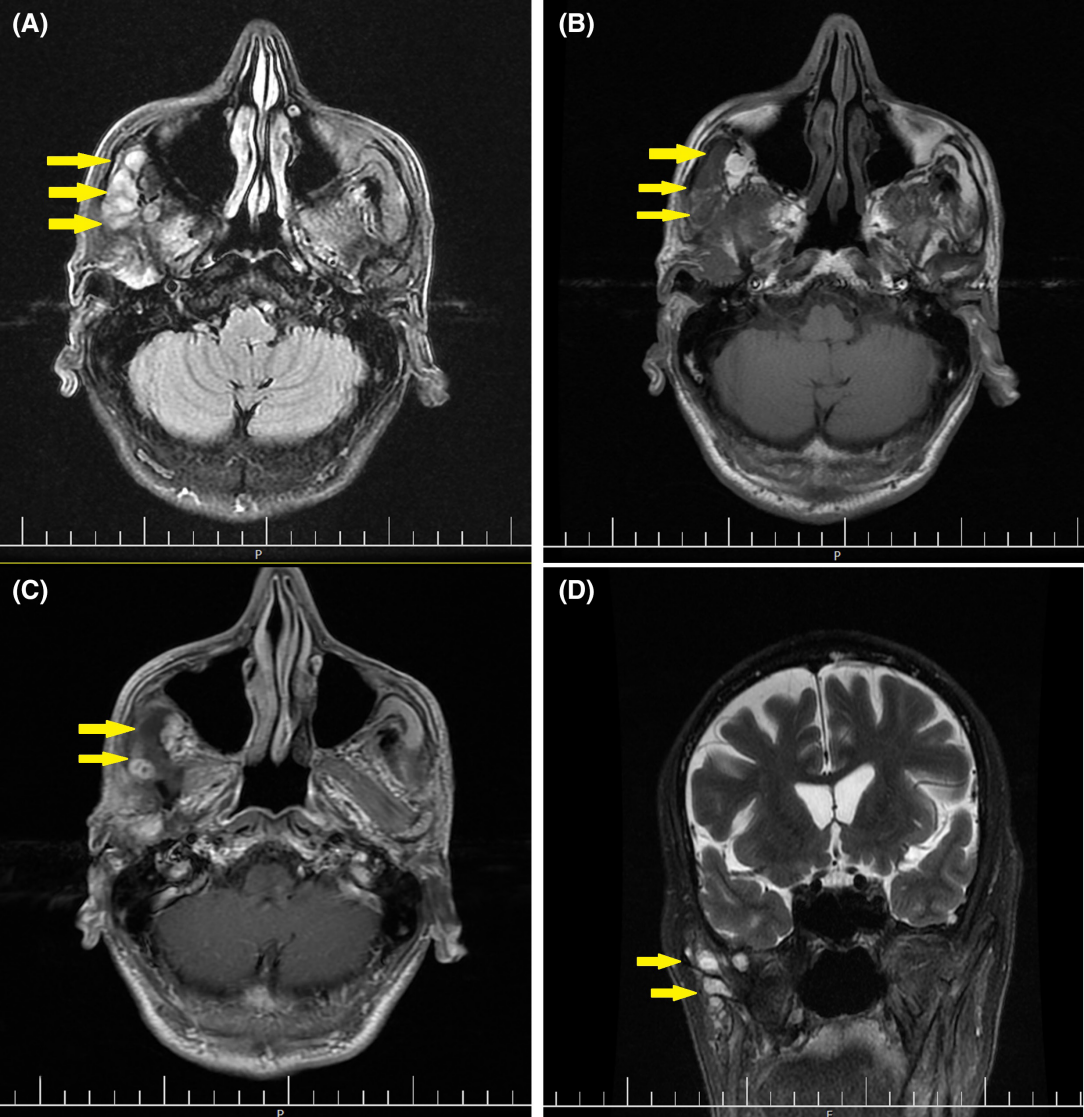
(A, B) Axial T2 and T1 weighted MRI demonstrate an expansile lytic lesion with cortical erosion and extraosseous extension in the right posterior region of the mandibular body and ramus. (C) Axial T1 weighted post‐contrast image shows homogeneous lesion enhancement with a few small non‐enhancing necrotic areas in the central parts. (D) Coronal T2 weighted and fat‐suppressed images reveal a large multilobulated aggressive mass in the right infratemporal fossa with involvement of the mandibular body and ramus (Yellow arrows indicate the lesion).

MRI Imaging demonstrated an expansile lytic mass lesion with cortical erosion and extraosseous extension in the right posterior region of the mandibular body and ramus. The mass was located between the masseter and pterygoid muscles, with no evidence of muscle invasion. Significant infiltration into the right mandibular body was observed, with the mandibular condyle involvement and extension to the inferior part of the temporalis fossa. Lymph node involvement of levels Ib and II was evident, with the largest lymph node measuring 16 × 10 mm at level Ib. After contrast administration, homogeneous enhancement of the mass lesion was observed, with few small non‐enhancing necrotic central parts (Figure 1). A tumoral invasion into the medullary space of the right side of the mandibular body and ramus was noted, along with extension into the right side of the oral cavity floor, inferior to the tongue. A 17 × 10 mm lymph node anterior to the right submandibular gland and a 21 × 12 mm enhancing lesion in the right cerebellopontine (CP) angle cistern were also detected.

**FIGURE 2:**
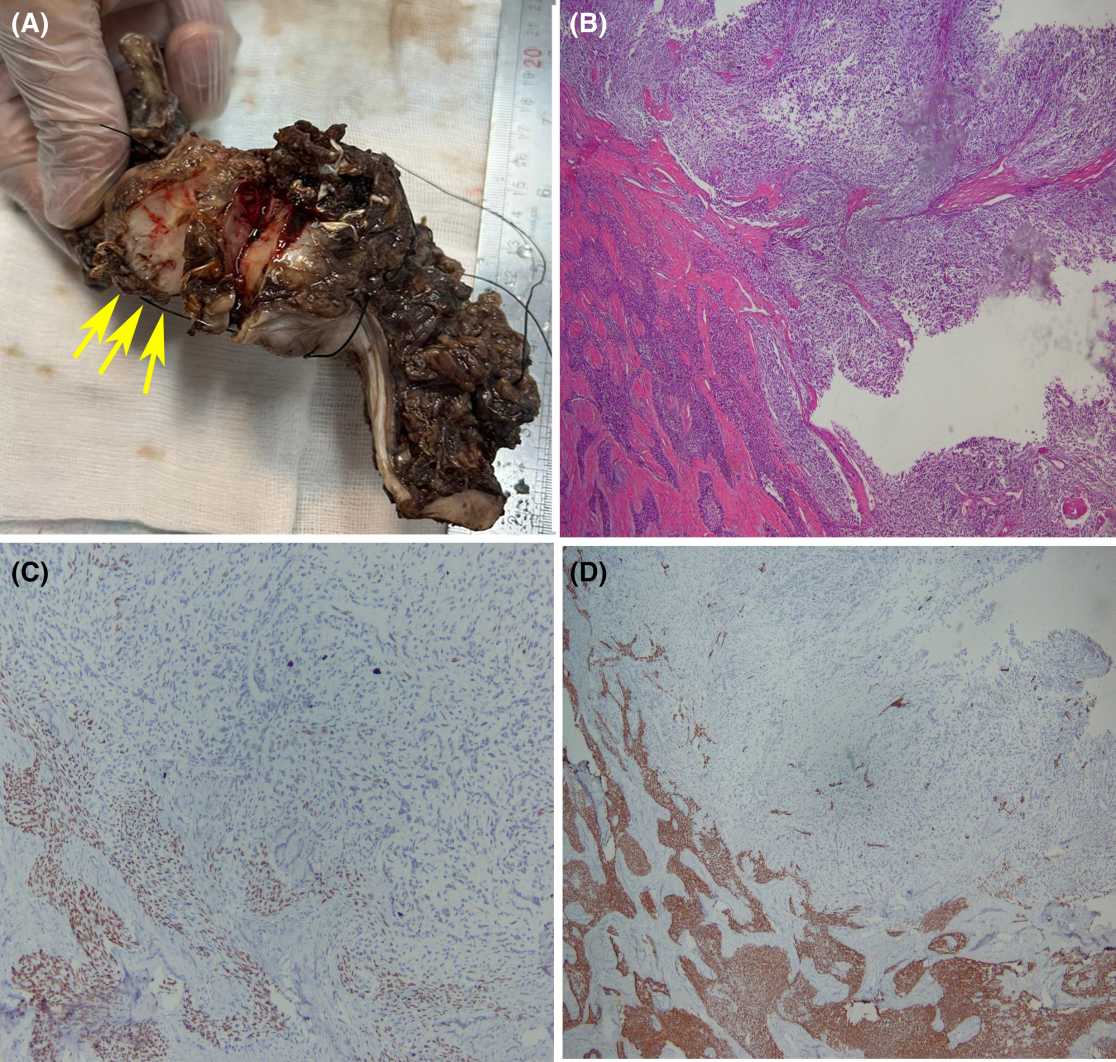
(A) Gross examination reveals a destructive lesion in the mandibular body with extension to the ramus. Cut sections show infiltrative behavior of the tumor to surrounding normal structures (Yellow arrow). (B) Hematoxylin and eosin (H&E)‐stained microscopic slides show neoplastic tissue composed of two separate populations; epithelial (left)‐ demonstrating odontogenic features including peripheral palisading and areas of reverse polarity in foci of peripheral palisaded epithelial cells is evident, and mesenchymal (right) cells (×40). (C, D) Immunohistochemical (IHC) study demonstrates positive nuclear staining for p63 (×100) and positive cytoplasmic staining for Pancytokeratin (AE_1_/AE_3_) (×40) in the epithelial component, but no immunoreactivity is seen in the mesenchymal component.

Under ultrasound (US) guidance, a core biopsy from the soft tissue mass on the right side of the mandible was performed. Microscopic examination of the excised lesion revealed two separate populations of neoplastic cells displaying prominent cellular atypia, nuclear pleomorphism, and numerous mitotic figures. Based on these findings, the final diagnosis was odontogenic carcinosarcoma (Figure 2). Based on the characteristics of the tumor in this case, the final American Joint Committee on Cancer (AJCC) staging classification is Stage IVA (T4 N1 M0).

### 3.3 Treatment

In November 2022, the patient underwent a right hemi‐mandibulectomy with resection of adjacent soft tissues, followed by a neck dissection encompassing levels I‐V due to lymph node involvement. The resultant defect was reconstructed using a pectoralis major flap without the incorporation of bony reconstruction. The treatment regimen was supplemented with adjuvant chemoradiotherapy. The patient was monitored with follow‐up visits at 3‐month intervals during the first year and 6‐month intervals during the second year.

### 3.4 Results

The lesion was excised during the initial post‐treatment phase, and the patient's recovery was uneventful. The most recent follow‐up MRI on July 2023 revealed no evidence of a mass or expansile lesion. This favorable outcome highlights the efficacy of our treatment approach. Importantly, no recurrence or metastasis was observed during the follow‐up period.

## 4 DISCUSSION

Whether odontogenic or non‐odontogenic, Carcinosarcoma poses a diagnostic challenge as it relies on morphological and immunohistochemical findings. Cytogenetic analysis is not typically helpful, especially in poorly differentiated tumors where immunohistochemical (IHC) studies are crucial in distinguishing epithelial from mesenchymal cell populations. Some researchers question the validity of the term “Carcinosarcoma,” suggesting that the mesenchymal component may represent an epithelial‐mesenchymal transition rather than a distinct entity. This perspective suggests that neoplastic cells could be high‐grade carcinoma with diverse microscopic features.

While the origin of the neoplasm as odontogenic or non‐odontogenic is a valid concern, if the diagnosis of Carcinosarcoma is accepted and clinical and radiographic findings support a primary intraosseous tumor, it is likely of odontogenic origin. Central Carcinosarcoma originating from odontogenic epithelial remnants in the jaws is more plausible than salivary gland tissues in the head and neck region.

Odontogenic carcinosarcoma (OCS) is an uncommon malignant odontogenic tumor with a biphasic structure, resembling the architectural features seen in ameloblastic fibroma. It is characterized by carcinomatous and sarcomatous components.^1^, ^4^, ^7^, ^8^ At present, the cause of OCS is not well understood. Research suggests that out of the cases studied, only 7 were primary lesions, with 10 potentially originating from primary lesion sites like ameloblastoma (AB), ameloblastic fibroma (AF), and ameloblastic fibrosarcoma (AFS) (Table 1),^5^, ^17^ this transformation's mechanism is not fully elucidated,^5^, ^14^, ^16^, ^17^ insufficient surgical resection and absence of adjuvant therapy,^5^, ^7^, ^14^, ^16^, ^17^ surgical trauma, and multiple surgical resections.^5^ Histopathological features of OCS closely resemble those of other odontogenic tumors (OTs), spanning from benign epithelial tumors to malignant tumors with metastatic potential, including recurrent ameloblastoma, ameloblastic carcinoma, and ameloblastic fibrosarcoma.^6^, ^8^, ^14^, ^18^

**TABLE 1:** Overview of clinical characteristics in documented instances of odontogenic carcinosarcoma.

First author (year of publication)	Age (years)/sex	Site/pre‐existing lesion	Follow‐up Period (years)	Mortality
Tahsinoglu (1981)^9^	30/F	Mandible/De novo	6 months	Survive
Tanaka (1991)^4^	63/M	Maxilla/Malignant Ameloblastoma	3.8	Death
Slater (1999)^10^	55/M	Mandible/De novo		Survive
Slama (2002)^11^	26/F	Mandible/Ameloblastic Fibrosarcoma	3	Death
Kunkel (2004)^12^	52/M	Mandible/Ameloblastic Fibrosarcoma	6	Death
DeLair (2007)^13^	19/F	Mandible/Ameloblastic Fibroma	2	Survive
Chikosi (2011)^14^	9/F	Mandible/Ameloblastoma	2.5	Death
Kim (2015)^6^	61/M	Mandible/De novo	2	Survive
Santos (2018)^15^	42/M	Maxilla/De novo	1	Survive
Soares (2019)^16^	22/M	Mandible/Ameloblastic Fibrosarcoma		Survive
Soares (2019)^16^	19/F	Mandible/De novo		Death
Salem (2020)^8^	28/M	Mandible/Premature Odontoma	9 months	Survive
Niu X (2021)^17^	58/F	Maxilla/Dentinoid	4 years	Survive
Abadi (2022)^5^	33/M	Mandible/Ameloblastic Fibroma	16 months	Survive
Majumdar (2022)^18^	24/M	Mandible/De novo		
NA Hasyim (2023)^19^	38/M	Mandible/Ameloblastoma		

Ameloblastoma (AB) is the second most prevalent benign epithelial odontogenic tumor. Slow‐growing nature of AB typically presents as a painless mass; however, it can sometimes exhibit aggressive behavior characterized by local invasiveness and bone resorption.^1^, ^2^ On the contrary, Ameloblastic carcinoma may originate de novo (primary type) or arise secondarily from initially benign ameloblastoma. Clinically, Ameloblastic carcinoma manifests as a rapidly growing, relatively painful tumor, often demonstrating aggressive cortical perforation and peripheral extension.^1^, ^2^, ^20^ Ameloblastic fibroma (AF) typically appears as asymptomatic, slow‐growing, and painless jaw swelling. These lesions are often incidentally discovered during routine radiographic examinations.^1^, ^2^ One of the primary differential diagnoses for odontogenic carcinosarcoma (OCS) is ameloblastic fibrosarcoma.^16^ AFS is a neoplasm that shares a similar structure with AF, comprising both epithelial and mesenchymal parts. However, AFS is distinguished by the presence of malignant proliferation in the mesenchymal component alone.^21^ Studies have reported that patients with AFS tend to be older, and lesions are more extensive compared to AF. Additionally, AFS and AF exhibit significant differences in clinical symptoms, bone expansion, adjacent tumor perforation, and recurrence rates. AFS is more commonly associated with multilocular radiolucency compared to AF.^21^

According to a review of previous studies, Odontogenic carcinosarcoma (OCS) has been noted in various age ranges, with an average patient age of 43.8 years, predominantly affecting males and the posterior mandibular region. Older males are more commonly affected.^7^, ^15^ Clinically, swelling and neurological symptoms can be seen in the patient. Radiographic assessments typically reveal multilocular radiolucency with indistinct borders and cortical perforation. This tumor demonstrates aggressive clinical behavior, with a high recurrence rate and a propensity for distant metastasis to the lung, lymph nodes, ribs, and pelvis.^7^

A comprehensive evaluation of histopathologic, immunohistochemistry, and clinical features is necessary to differentiate odontogenic carcinosarcoma from other related diseases. In cases where OCS is detected early, and displays mild features of malignancy, adequate surgical excision alone may lead to favorable outcomes. However, based on previous reports, the main treatment approach, akin to other malignant odontogenic tumors, typically entails surgical excision by a wide margin of safety and neck dissection. The potential benefit of adjunctive radiotherapy and chemotherapy in soft tissue invasion cases is a topic of debate but could be advantageous.^7^ In previous cases, the majority of patients have undergone radical resection, with some also receiving adjunctive chemotherapy and radiotherapy. However, the optimal treatment approach is yet to be determined. Further case reports and long‐term follow‐up studies are necessary better to understand odontogenic carcinosarcoma and its corresponding survival rates.

## 5 CONCLUSION

Upon histopathological examination, we present a mandibular case of odontogenic carcinosarcoma, displaying malignant characteristics in both the epithelial and ectomesenchymal components. Despite an unclear etiology, these tumors manifest aggressive clinical behavior, marked by high rates of recurrence and metastasis. Extended follow‐up periods cannot be overstated, as they are crucial to unravel odontogenic carcinosarcoma's nature and survival outcomes, emphasizing the need for ongoing research and vigilance.

Given the challenging prognosis, surgical removal of a portion of the mandible emerges as the most effective treatment strategy. Early detection and aggressive surgical management significantly influence patient outcomes, although recurrence remains a significant hurdle. Therefore, the importance of continued research and long‐term follow‐up studies cannot be overstated in our quest to understand this rare and aggressive tumor better.

## AUTHOR CONTRIBUTIONS

**Pouyan Aminishakib:** Conceptualization; data curation; formal analysis; investigation; project administration; resources; supervision; validation; visualization. **Farzaneh Mosavat:** Conceptualization; data curation; formal analysis; project administration; software; supervision; writing – review and editing. **Mahsa Bayati:** Conceptualization; data curation; formal analysis; investigation; project administration; resources; validation; visualization; writing – original draft; writing – review and editing. **Ata Garajei:** Conceptualization; data curation; funding acquisition; methodology; resources; software; supervision; validation.

## FUNDING INFORMATION

This article received no particular funding from any organizations.

## CONFLICT OF INTEREST STATEMENT

The authors verified no conflicts of interest.

## ETHICS STATEMENT

Ethical approval from our institution was not required to report individual cases.

## CONSENT

Written informed consent was obtained from the patient to publish this report in accordance with the journal's patient consent policy.

## Data Availability

The data supporting this study can be obtained from the corresponding author upon a reasonable request.
